# OM-MSCs Alleviate the Golgi Apparatus Stress Response following Cerebral Ischemia/Reperfusion Injury via the PEDF-PI3K/Akt/mTOR Signaling Pathway

**DOI:** 10.1155/2021/4805040

**Published:** 2021-11-13

**Authors:** Jialin He, Jianyang Liu, Yan Huang, Xiangqi Tang, Han Xiao, Zuo Liu, Zheng Jiang, Liuwang Zeng, Zhiping Hu, Ming Lu

**Affiliations:** ^1^Department of Neurology, The Second Xiangya Hospital, Central South University, Changsha, 410011 Hunan, China; ^2^National Health Commission Key Laboratory of Birth Defect for Research and Prevention, Hunan Provincial Maternal and Child Health Care Hospital, Changsha, 410008 Hunan, China; ^3^Department of Neurosurgery, Second Affiliated Hospital of Hunan Normal University, Changsha, 410003 Hunan, China; ^4^Hunan Provincial Key Laboratory of Neurorestoratology, Second Affiliated Hospital of Hunan Normal University, Changsha, 410003 Hunan, China

## Abstract

The mechanism of Golgi apparatus (GA) stress responses mediated by GOLPH3 has been widely studied in ischemic stroke, and the neuroprotection effect of olfactory mucosa mesenchymal stem cells (OM-MSCs) against cerebral ischemia/reperfusion injury (IRI) has been preliminarily presented. However, the exact role of OM-MSCs in the GA stress response following cerebral IRI remains to be elucidated. In the present study, we used an oxygen-glucose deprivation/reoxygenation (OGD/R) model and reversible middle cerebral artery occlusion (MCAO) model to simulate cerebral IRI in vitro and in vivo. Our results showed that the level of GOLPH3 protein, reactive oxygen species (ROS), and Ca^2+^ was upregulated, SPCA1 level was downregulated, and GA fragmentation was increased in ischemic stroke models, and OM-MSC treatment clearly ameliorated these GA stress responses in vitro and in vivo. Subsequently, the knockdown of PEDF in OM-MSCs using *PEDF*-specific siRNA further demonstrated that secretion of PEDF in OM-MSCs protected OGD/R-treated N2a cells and MCAO rats from GA stress response. Additionally, rescue experiment using specific pathway inhibitors suggested that OM-MSCs could promote the phosphorylation of the PI3K/Akt/mTOR pathway, thereby mitigating OGD/R-induced GA stress response and excessive autophagy. In conclusion, OM-MSCs minimized the GA stress response following cerebral IRI, at least partially, through the PEDF-PI3K/Akt/mTOR pathway.

## 1. Introduction

Ischemic stroke is a common disease with high morbidity, mortality, and disability rates [1]. Brain injury caused by ischemic stroke involves intricate pathophysiological processes, including oxidative stress, calcium overload, and inflammation, which not only are directly attributed to the interruption of cerebral blood flow but are also inseparable from the subsequent recanalization [2]. Therefore, the inhibition of cerebral ischemia/reperfusion injury (IRI) is of great significance for the treatment of ischemic stroke.

As one of the early events of cerebral IRI, oxidative stress contributes to the catastrophe of brain tissue together with other detriment factors [3], while therapeutic options targeting oxidative stress are conducive to the attenuation of cerebral injury and reconstruction of neural circuits. It is initially recognized that only the mitochondria and endoplasmic reticulum are involved in the process of oxidative stress; however, in the past decade, accumulating evidence demonstrates the participation of the Golgi apparatus (GA) in oxidative stress, which is denoted as “GA stress” [4, 5]. GA can trigger downstream signals and induce GA fragmentation, intracellular Ca^2+^ overload, and apoptosis by regulating the activity of Golgi-resident secretory pathway Ca^2+^-ATPase (SPCA) and altering the membrane surface components under oxidative stress. Previous reports have suggested the existence of GA stress response in ischemic stroke where inhibition of these responses promoted functional recovery [6, 7].

Furthermore, Golgi phosphoprotein 3 (GOLPH3, also known as GPP34/GMx33/MIDAS), a GA stress-associated protein, is a peripheral membrane protein of the trans-Golgi network that is located on the cytoplasmic surface of the trans-Golgi [8, 9]. GOLPH3 is stimulated by oxidative stress and is involved in signal transduction [10], while GOLPH3 silencing contributed towards alleviating GA stress response. Therefore, targeting GA stress mediated by GOLPH3 would offer an appealing strategy for ameliorating cerebral IRI.

Since the existing vascular recanalization therapies for acute ischemic stroke are time-bounded [11, 12], the search for alternative therapeutic options, such as stem cell-based therapy, has gradually attracted attention and has gained considerable success [13]. Of note, the easy accessibility and pleiotropic effects make mesenchymal stem cells (MSCs) an ideal cell source for ischemic stroke therapies [14]. Olfactory mucosa mesenchymal stem cells (OM-MSCs), localized in the lamina propria of the olfactory mucosa, display the advantages of convenient accessibility and high proliferation efficiency and enable autologous transplantation over other types of MSCs [15, 16]. Moreover, the neural crest origin of OM-MSCs makes them a suitable candidate for the treatment of neurological diseases [17, 18]. Notably, several recent studies have demonstrated the neuroprotective effect of OM-MSCs against cerebral IRI [19–21], one of which preliminarily suggested OM-MSC-mediated restoration of SPCA1 expression and reduction of GA dissolution in ischemic stroke rats [21]. However, evidence regarding the exact mechanism of OM-MSCs in GA stress response following cerebral IRI remains lacking.

The secretome of MSCs is gradually being recognized to be more pronounced than the cell replacement effect in ischemic stroke, due to the inability of MSCs to penetrate the blood-brain barrier [22]. The secretome of OM-MSCs has been investigated by Ge et al. [23] who reported that pigment epithelium-derived factor (PEDF), which is a class of multifunctional proteins that are abundant in brain tissue [24] and are related to neurotrophy, was one of the secreted proteins of OM-MSCs. Because PEDF promotes the phosphorylation of Akt [25] and the activation of the PI3k/Akt/mTOR pathway plays a crucial role in the metabolic regulation, it was hypothesized that OM-MSCs may alleviate the GA stress response after cerebral IRI by activating the PI3K/Akt/mTOR pathway through secretion of PEDF.

In this study, we aim to understand the role of OM-MSCs in GA stress responses following cerebral IRI and elaborate on the possible mechanism. In summary, the present study demonstrates that targeting the GA will be one of the mechanisms by which OM-MSCs exert neuroprotective effects in ischemic stroke.

## 2. Materials and Methods

### 2.1. Ethics Statement

OM-MSCs were obtained from healthy donors (two males, 27 years old and 33 years old, respectively) at the Second Affiliated Hospital of Hunan Normal University, Changsha, China. A human nasal mucosa biopsy was performed by otolaryngology endoscopy operation at the Department of Otolaryngologic Surgery, the Second Affiliated Hospital of Hunan Normal University. All procedures were approved by the Ethics Committee of the Hunan Normal University. All volunteers signed the informed consent for the use of intranasal biopsy samples for research in accordance with the Helsinki convention (1964).

### 2.2. Human OM-MSC Isolation and Culture

Human OM-MSCs were isolated and cultured according to a previous published protocol [23]. Olfactory tissue samples were obtained from the root of the medial aspect of the middle turbinate, washed 3 times with antibiotic-antimycotic solution (Invitrogen, CA), and then cut into 1 mm^3^ to 2 mm^3^ pieces with a thickness ranging from 200 to 500 *μ*m. The pieces were then cultured in Dulbecco's modified Eagle's medium: nutrient mixture F12 (DMEM/F12; Invitrogen, CA) containing 10% fetal bovine serum (FBS; Gibco, Australia) and incubated at 37°C in 5% CO_2_. The culture medium was changed every 3 or 4 days. OM-MSCs at passages 3 and 4 were used for further experiments. The characteristics of the OM-MSCs were identified using PE-conjugated antibodies against specific membrane markers (CD34, CD45, CD73, CD90, and CD105; eBioscience, San Diego, CA, USA) by using a flow cytometer (Beckman, USA).

### 2.3. Oxygen-Glucose Deprivation/Reoxygenation (OGD/R) Induction

Mouse N2a cells were purchased from the Cell Storage Center of Chinese Academy of Sciences (Shanghai, China) and cultured in DMEM (Invitrogen, CA, USA) containing 10% FBS at 37°C in 5% CO_2_.

The cerebral IRI in vitro model was established by OGD/R as described previously [21]. N2a cells were placed in a modular incubator chamber (Billups Rothenberg, Inc., Del Mar, CA) with a gas mixture of 5% CO_2_ and 95% N_2_. The culture medium was replaced with deoxygenated glucose-free Hanks' Balanced Salt Solution (Biological Industries, Israel). After 4 h of OGD, the N2a cells were maintained in DMEM without FBS and incubated under normoxic conditions for 24 h. For the normal group, N2a cells were cultured in DMEM containing 10% FBS under normoxic condition.

### 2.4. Coculture of OM-MSCs and N2a Cells

The coculture system was set up via the 0.4 mm pore size Transwell plates (Corning, USA). 1 × 10^5^ N2a cells grown in 6-well plates were subjected to OGD treatment as described above. At the same time when reoxygenation began, the N2a cells were rescued by the addition of 1 × 10^5^ OM-MSCs, OM-MSC^NCsiRNA^, or OM-MSC^PEDFsiRNA^ to the Transwell membrane inserts and incubated for 24 h. DMEM without FBS was used during reoxygenation.

### 2.5. Rat Reversible Middle Cerebral Artery Occlusion (MCAO) Model

All animal procedures were approved by the Laboratory Animal Ethics Committee of the Second Affiliated Hospital of Hunan Normal University. All experimental procedures were performed in accordance with the Guide for the Care and Use of Experimental Animals. Male Sprague–Dawley rats (SD rats) weighing 250–300 g were housed under controlled housing conditions with a 12 h light/dark cycle with food and water ad libitum.

The right MCAO model was carried out as described in previous studies [21, 26]. Rats were initially anesthetized with 3.5% isoflurane and maintained with 1.0–2.0% isoflurane under 2 : 1 N_2_O/O_2_ using a face mask. The right common carotid artery, internal carotid artery, and external carotid artery (ECA) were separated, and an incision was made in the right common carotid artery using ophthalmic scissors. A surgical filament (0.26 mm diameter; Beijing Cinontech Co. Ltd., China) was inserted into the internal carotid artery, with the length of the line being 18–20 mm. After 120 min of ischemia, the filament was withdrawn for reperfusion. Rats in the sham operation group underwent the same procedure without the insertion of the filament.

In total, 40 adult male SD rats were randomly divided into four groups: sham operation group, MCAO+saline group, MCAO+OM-MSC^NCsiRNA^ group, and OM-MSC^PEDFsiRNA^ group (*n* = 10 animals per group). Specifically, the inclusion and exclusion criteria of the MCAO+saline, MCAO+OM-MSC^NCsiRNA^, and OM-MSC^PEDFsiRNA^ groups were based on the Zea-Longa score when the rats were awake after surgery [26]. SD rats with a score of 1 to 3 were included in the subsequent experiment, while SD rats that died, or those with a score of 0 and 4, were dropped. To compensate for dropouts, two additional rats were enrolled resulting in an overall study population of 42 rats. The dropout animals were euthanized by anesthetization with 3.5% isoflurane and decapitation.

### 2.6. OM-MSC Transplantation

In the MCAO+OM-MSC^NCsiRNA^ group and MCAO+OM-MSC^PEDFsiRNA^ group, the rats received a tail vein injection of 5.0 × 10^6^ OM-MSC^NCsiRNA^ or OM-MSC^PEDFsiRNA^ dissolved in 1 ml saline at 24 h after MCAO model induction, while the rats received tail vein injection of 1 ml saline in the MCAO+saline group. At seven days after reperfusion, rats in each group were euthanized through anesthetization with 3.5% isoflurane and decapitation, and brain samples were collected for the experiment.

### 2.7. Hematoxylin-Eosin (H&E) Staining

Animals were anesthetized with 3.5% isoflurane and underwent transcardiac perfusion with 4% paraformaldehyde. Appropriate weight brain tissues were immersed in 4% paraformaldehyde for 24 h and embedded in paraffin. Subsequently, the sections were deparaffinized with graded ethanol and xylene, and 4 *μ*m sections were prepared for hematoxylin-eosin staining. After dehydration with graded ethanol and xylene, the histological structure of the brain tract was observed under a light microscope (Motic, BA210T, China).

### 2.8. Behavioral Test

Functional behaviors in rats were tested at days 0, 3, and 7 post-MCAO. All behavioral tests were conducted by two investigators who were blinded to the experimental groups. The 18-point modified neurologic severity scores (mNSS) were used to evaluate neurological function [21]. A higher score represented a more severe neurological deficit.

### 2.9. Apoptosis Assay

The apoptosis percentage of N2a cells was monitored using a FITC-Annexin V apoptosis detection kit (KeyGen Biotech, Jiangsu, China). 500 *μ*l cells was incubated with 5 *μ*l FITC-Annexin V detection kit solution for 10 min at room temperature in the dark. The rates of cell apoptosis were recorded using a flow cytometer (Beckman, CA, USA).

### 2.10. Measurement of Intracellular Reactive Oxygen Species (ROS) Generation

Intracellular ROS levels of N2a cells and the ipsilateral cortex of SD rats were measured using an oxidation-sensitive fluorescent probe (DCFH-DA) kit (Beyotime, Shanghai, China). The N2a cells or digested brain tissue was loaded with DCFH-DA at 10 *μ*M in all wells. After further culture for 20 min at 37°C in the dark, the cells were then washed three times with serum-free medium and resuspended in PBS. Fluorescence was measured by using a flow cytometer (Beckman, CA, USA).

### 2.11. Lipid Peroxidation (LPO) Measurement

The ipsilateral cortex of SD rats from each group was used for the LPO measurements. LPO level was detected using the lipid peroxidation assay kit (Nanjing Jiancheng Bioengineering Institute, Jiangsu, China). The brain tissues were digested and then incubated with lipid peroxidation assay kit solution at 45°C for 1 h. After centrifugation, the supernatant was used to determine the optical density (OD) value under a spectrophotometer (Tianpu, Shanghai, China). LPO concentration was calculated based on the protein concentration and OD value according to the manufacturer's instructions.

### 2.12. Analysis of Intracellular Ca^2+^ Concentration

Intracellular Ca^2+^ concentration of N2a cells and the ipsilateral cortex of SD rats was detected in Fluo-3/AM- (Beyotime, Shanghai, China) loaded cells by flow cytometry. The N2a cells or digested brain tissues were incubated with 5 mM Fluo-3/AM (Beyotime) at 37°C for 0.5 h. After washing and resuspension in PBS, intracellular Ca^2+^ level was measured at an excitation wavelength of 488 nm and an emission wavelength of 530 nm using a flow cytometer (Beckman, CA, USA).

### 2.13. Western Blotting

N2a cells, the ipsilateral cortex of SD rats, and OM-MSCs were processed for western blotting as described previously. Proteins were transferred to the PVDF membrane (Millipore, USA) when the bromophenol blue reached the bottom, and the blots were blocked in 4% BSA in TBST (0.05%) solution for 1 h at room temperature and then incubated at 4°C overnight with the corresponding primary antibody. After incubation with secondary antibodies at room temperature for at least 1 h, the blot was visualized by the ChemiDoc XRS imaging system.

The primary antibodies were as follows: anti-caspase3 (19677-1-AP, Proteintech, USA), anti-GOLPH3 (ab98023, Abcam, Cambridge, UK), anti-SPCA1 (ab126171, Abcam, Cambridge, UK), anti-LC3 (ab192890, Abcam, Cambridge, UK), anti-LAMP1 (ab208943, Abcam, Cambridge, UK), anti-Akt (60203-2-Ig, Proteintech, Chicago, USA), anti-p-Akt (ab38449, Abcam, Cambridge, UK), anti-mTOR (20657-1-AP, Proteintech, Chicago, USA), anti-p-mTOR (ab109268, Abcam, Cambridge, UK), anti-PEDF (bs-20784R, Bioss, Beijing, China), and anti-*β*-actin (66009-1-Ig, Proteintech, Chicago, USA). The anti-rabbit and anti-mouse IgG secondary antibodies were obtained from Proteintech (Chicago, USA).

### 2.14. Immunofluorescence Analysis

The expression of LC3 and SPCA1 was also evaluated by immunofluorescence analysis. After blocking, N2a cells were incubated in the primary antibody against LC3 (14600-1-AP, Proteintech, USA, dilution 1 : 50) or SPCA1 (ab126171, Abcam, Cambridge, UK, dilution 1 : 50) overnight at 4°C. Next, N2a cells were incubated with goat anti-rabbit IgG(H+L) (SA00013-2, Proteintech, USA, dilution 1 : 200) for 90 min at room temperature and then stained with DAPI (Wellbio, Changsha, China). The slides were observed under a fluorescent microscope (Motic, China).

### 2.15. Transmission Electron Microscope

N2a cells were fixed in 4% paraformaldehyde and 2% glutaraldehyde in 0.1 M PBS at pH 7.4, overnight at 4°C. After osmium tetroxide postfixation and alcohol dehydration, the samples were embedded in epoxy resin, and the embedded fragments were sliced and stained with uranyl acetate and lead citrate and viewed under a transmission electron microscope (Hitachi HT7700, Japan) at 80 kV. The number of autophagosomes and autolysosomes was evaluated by randomly selecting five micrographs per sample.

### 2.16. Pharmacological Intervention

Perifosine (A8309, APExBIO, USA, 10 *μ*M, dissolved in PBS) and rapamycin (A8167, APExBIO, USA, 50 nM, dissolved in PBS) were used to inhibit Akt and mTOR, respectively. In the corresponding treatment group, each drug was added to the growth medium of N2a cells at the onset of reoxygenation.

### 2.17. Real-Time qRT-PCR

Total RNA was isolated from OM-MSCs using the TRIzol reagent (Thermo Fisher Scientific, USA). The UltraSYBR Mixture (Beijing ComWin Biotech Co., Ltd., China) was used to perform qRT-PCR according to the manufacturer's instructions. The following qPCR primer sequences were used to generate specific fragments: 5′-TATGACTTGATCAGCAG-3′ and 5′-AGCTTCATCTCCTGCAGGGA-3′ for human PEDF and 5′-ACCCTGAAGTACCCCATCGAG-3′ and 5′-AGCACAGCCTGGATAGCAAC-3′ for human *β*-actin. The mRNA expression level of PEDF was normalized to *β*-actin expression, and relative expression was calculated using the standard 2^−ΔΔCt^ method.

### 2.18. Enzyme-Linked Immunosorbent Assay (ELISA)

PEDF levels in the cell culture supernatants of OM-MSCs were detected using a human PEDF ELISA kit (CSB-E08818h, Cusabio, China) according to the manufacturer's protocols. Samples in each well were firstly incubated with working solution and then reacted with the substrate solution at 37°C for 0.5 h. The absorbance was recorded at 450 nm using a microplate reader (Themo Fisher, USA) after the termination solution was added.

### 2.19. Small Interfering RNA (siRNA) Knockdown of PEDF

For siRNA knockdown, the siRNA target sequence 5′-TCACCAGACTTTAGCAAGA-3′ was selected. PEDF expression in OM-MSCs was silenced with PEDF siRNA using a siRNA transfection kit (Ribobio Co., Ltd, Guangzhou, China), according to the manufacturer's instruction. The efficiency of PEDF knockdown in OM-MSCs was verified by qRT-PCR, ELISA, and western blotting.

### 2.20. Statistical Analysis

Statistical analyses were performed using SPSS statistical software (SPSS 22.0, Inc., Chicago, IL, USA). After testing for normal distribution, the data of two independent variables were analyzed using the Student *t*-test. For three or more variables, one-way analysis of variance (ANOVA) was performed, followed by post hoc analysis using Tukey's test. A two-way ANOVA was used to analyze the behavior test data. All data were presented as the mean ± standard deviations (SD) based on three independent experiments. Differences between the mean values were considered significant at a *p* value < 0.05.

## 3. Results

### 3.1. Identification of OM-MSCs

Under light microscopy, OM-MSCs adhered to the surface of the culture plate and adopted a fibroblastic or spindle-shaped morphology (Supplementary Figure S1A). The immunophenotype of OM-MSCs exhibited positive expression of CD73, CD90, and CD105 and negative expression of CD34 and CD45 (Supplementary Figure S1B).

### 3.2. OM-MSCs Ameliorated the GA Stress Response in OGD/R-Treated N2a Cells

**Figure 1:**
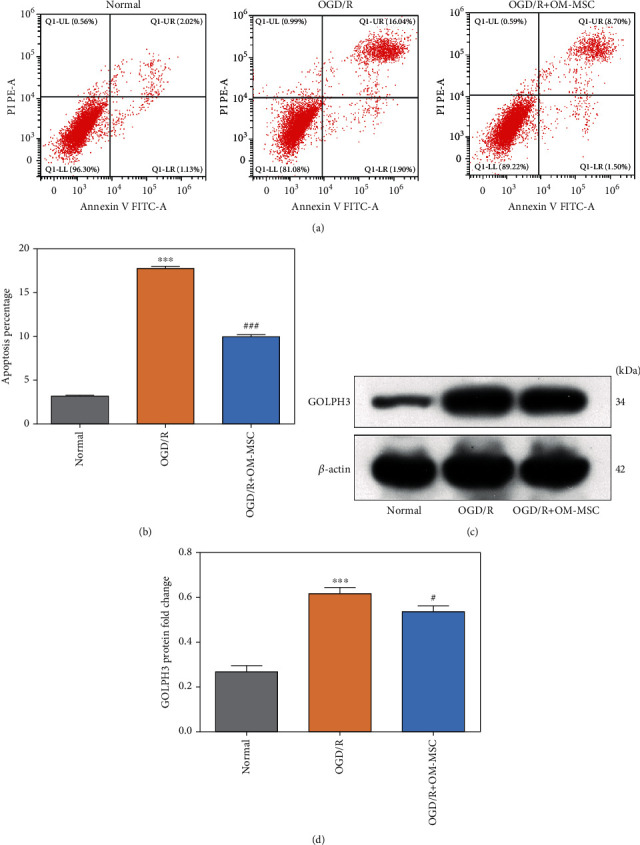
Olfactory mucosa mesenchymal stem cells (OM-MSCs) ameliorated the GA stress response in OGD/R- (oxygen and glucose deprivation/reoxygenation-) treated N2a cells. (a, b) Apoptosis percentage was evaluated by flow cytometry analysis. (c, d) The protein expression of GOLPH3 was detected by western blotting. OGD/R: oxygen and glucose deprivation for 4 h and then reoxygenation for 24 h. Data were displayed as mean ± SD based on three independent experiments. ^∗∗∗^*p* < 0.001 compared with the normal group; ^#^*p* < 0.05, ^###^*p* < 0.001 compared with the OGD/R group.

N2a cells were subjected to OGD/R insult to mimic cerebral IRI in vitro and then cocultured with OM-MSCs to explore whether OM-MSCs could attenuate cerebral IRI. The results demonstrated that the apoptosis rate was significantly upregulated in the OGD/R group as compared to the normal group, and these were notably diminished after treatment with the OM-MSC coculture (Figures 1(a) and 1(b)).

Moreover, the GA stress-associated protein GOLPH3 levels of OGD/R-treated N2a cells were significantly higher than those of the normal group, which were reversed by OM-MSC coculture (Figures 1(c) and 1(c)). Therefore, the above findings suggested that coculture with OM-MSCs contributed to the amelioration of GA stress response in OGD/R-treated N2a cells.

### 3.3. OM-MSCs Alleviated the OGD/R-Induced GA Stress Response in N2a Cells through PEDF Production

**Figure 2:**
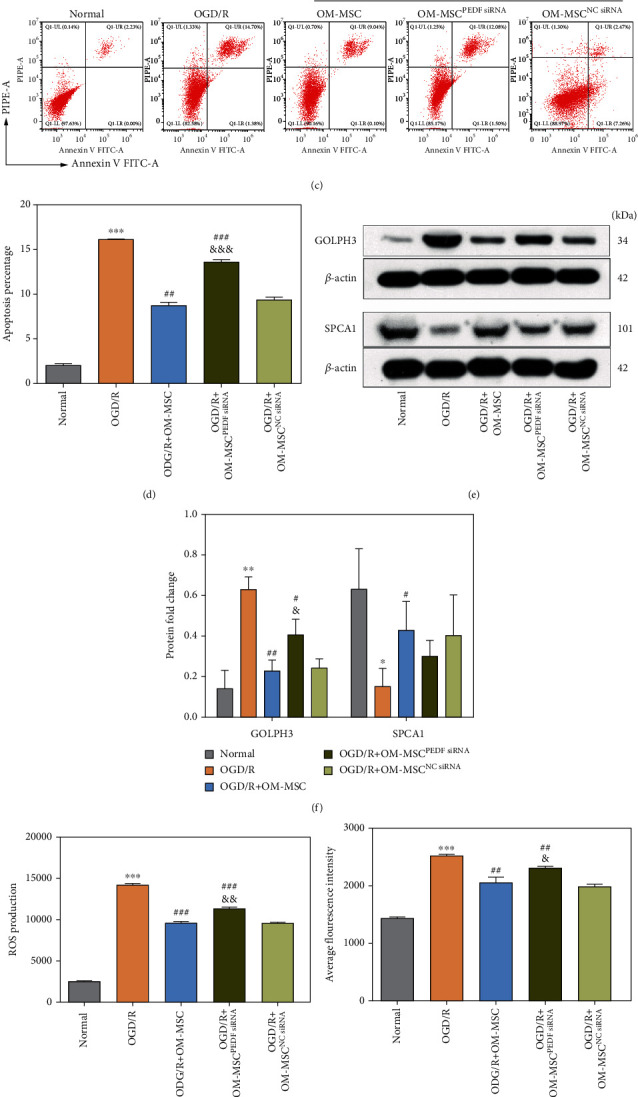
OM-MSCs alleviated the OGD/R-induced GA stress response in N2a cells through PEDF production. (a, b) The protein level of PEDF in N2a cells was measured by western blotting. (c, d) Apoptosis percentage was evaluated using flow cytometry analysis. (e, f) The protein expression of GOLPH3 and SPCA1 was determined using western blotting. (g) Intracellular ROS level was measured by flow cytometry analysis using an oxidation-sensitive fluorescent probe (DCFH-DA). (h) Intracellular Ca^2+^ concentration in N2a cells was examined by flow cytometry analysis using a Fluo-3/AM kit. (i) The representative image of GA ultramicrostructure changes by using a transmission electron microscope (scale bar = 2.0 *μ*m). The GA was indicated by the red arrow. Data were displayed as mean ± SD based on three independent experiments. ^∗^*p* < 0.05, ^∗∗^*p* < 0.01, and ^∗∗∗^*p* < 0.001 compared with the normal group; ^#^*p* < 0.05, ^##^*p* < 0.01, and ^###^*p* < 0.001 compared with the OGD/R group; ^&^*p* < 0.05, ^&&^*p* < 0.01, and ^&&&^*p* < 0.001 compared with the OGD+OM-MSC group.

Previous data indicated that PEDF protein was secreted in OM-MSCs, and the loss-of-function assay of PEDF in OM-MSCs was further performed to determine the specific molecular mechanism of OM-MSC-mediated regulation of GA stress response in N2a cells following OGD/R insult. First, the protein levels of PEDF in N2a cells were found to be reduced after OGD/R insult in comparison to the normal group, and OM-MSCs were able to upregulate the PEDF levels in N2a cells after OGD/R injury (Figures 2(a) and 2(b)). Subsequently, PEDF-specific siRNA was constructed and transfected into OM-MSCs to inhibit >the production of PEDF in OM-MSCs, and the results obtained by PCR, western blotting, and ELISA showed that *PEDF* siRNA contributed to a notable decrease in the PEDF mRNA and protein levels compared with control siRNA (Supplementary Figure S2A-D). Moreover, *PEDF* silencing in OM-MSCs limited the ability of OM-MSCs to inhibit the apoptosis rate (Figures 2(c) and 2(d)) and hampered the OM-MSC-mediated mitigation of OGD/R-induced GA stress responses. More precisely, a distinct elevation in the GOLPH3 expression level, ROS level, and Ca^2+^ concentration was observed in the OM-MSC coculture group after *PEDF* knockdown (Figures 2(e)–2(h)), and the upregulation of SPCA1 in the coculture group could also be blocked by *PEDF* silencing in OM-MSCs, but the results were not statistically significant (Figures 2(e) and 2(f)). Furthermore, as depicted in Figure 2(i), the morphology of GA in the OGD/R group displayed prominent alterations, including edema and dissolution, compared to that in the normal group. OM-MSC coculture alleviated GA fragmentation induced by OGD/R insult, while this beneficial effect could be neutralized by the *PEDF* silencing. In conclusion, OM-MSCs minimized the OGD/R-induced GA stress response through the production of PEDF.

### 3.4. OM-MSCs Attenuated the GA Stress Response in MCAO Rats by Secreting PEDF

**Figure 3:**
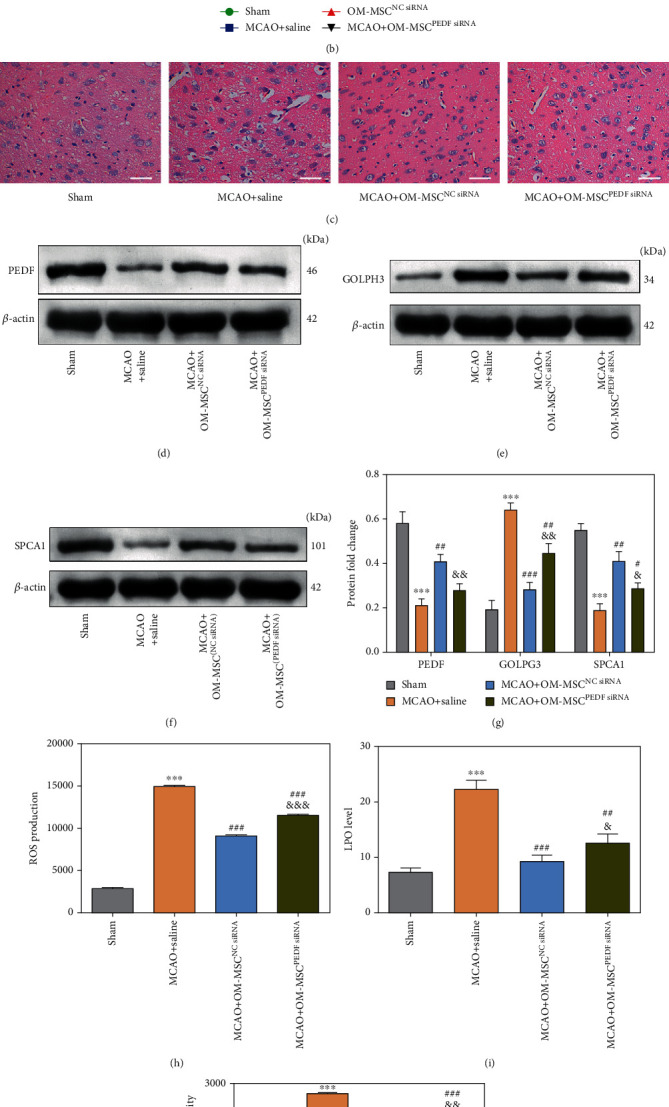
OM-MSCs attenuated the GA stress response in MCAO rats by secreting PEDF. (a) Schematic diagram of the experimental design for in vivo experiments. (b) The modified neurological severity score (mNSS) test was performed at 0 (pre-MCAO), 3, and 7 days after MCAO operation (*n* = 10 animal per group). (c) The pathologic changes of brain tissues were assessed using H&E staining (scale bar = 100 *μ*m). (d–g) The protein levels of PEDF, GOLPH3, and SPCA1 in ipsilateral brain samples of rats were measured by western blotting. (h) The ROS production of ipsilateral brain samples was measured by flow cytometry analysis using an oxidation-sensitive fluorescent probe (DCFH-DA). (i) The lipid peroxidation (LPO) levels of ipsilateral brain samples were measured by using a LPO assay kit. (j) The Ca^2+^ concentration of ipsilateral brain samples was examined by flow cytometry analysis using a Fluo-3/AM kit. OM-MSC^PEDFsiRNA^: knockdown of PEDF in OM-MSCs by transfecting PEDF-specific siRNA; OM-MSC^NCsiRNA^: OM-MSCs were transfected with normal control siRNA. Data were displayed as mean ± SD (*n* = 3 animals per group except for mNSS). ^∗∗∗^*p* < 0.001 compared with the sham group; ^#^*p* < 0.05, ^##^*p* < 0.01, and ^###^*p* < 0.001 compared with the MCAO+saline group; ^&^*p* < 0.05, ^&&^*p* < 0.01, and ^&&&^*p* < 0.001, compared with the MCAO+OM-MSC^NCsiRNA^ group.

MCAO rat models were established to recognize the neuroprotection mechanism of OM-MSCs against the GA stress response in vivo (Figure 3(a)). It was found that, compared to the sham group, the MCAO+saline group had significantly elevated mNSS at 3 days after the MCAO procedure. However, on day 7, the transplantation of OM-MSCs markedly lowered the mNSS levels in the OM-MSC^NCsiRNA^ and OM-MSC^PEDFsiRNA^ groups. PEDF ablation weakened the effect of OM-MSCs on mNSS at 7 days after MCAO operation compared to the OM-MSC^NCsiRNA^ group (Figure 3(b)). Additionally, OM-MSC transplantation reduced the pathological damage in MCAO rats (Figure 3(c)). OM-MSC administration upregulated PEDF and SPCA1 expression and downregulated GOLPH3 expression in MCAO rats (Figures 3(d)–3(g)). OM-MSCs also repressed the enhancement of intracellular ROS, LPO, and Ca^2+^ concentration in the MCAO group (Figures 3(h)–3(j)).

The effects of OM-MSCs were further identified by silencing PEDF in OM-MSCs. Concretely, despite no effect on the pathological damage, the advantageous impact of OM-MSCs on the GA stress response could be partially abrogated by *PEDF* siRNA, as evidenced by the alterations in protein expression of GOLPH3 and SPCA1 (Figures 3(e)–3(g)), ROS production, the LPO level, and Ca^2+^ concentration (Figures 3(h) and 3(i)). The above data illustrated that OM-MSCs attenuated the GA stress response in MCAO rats partially by secreting PEDF.

### 3.5. OM-MSCs Activated the PI3K/Akt/mTOR Pathway via PEDF

**Figure 4:**
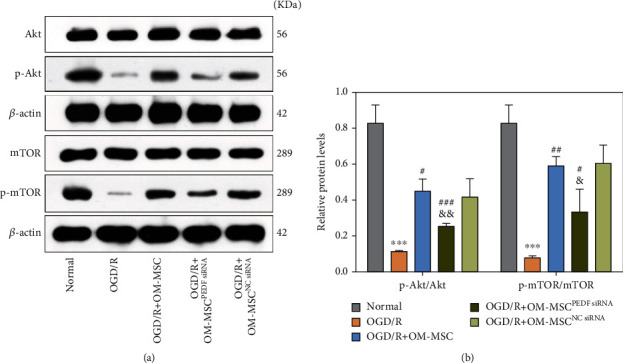
OM-MSCs promoted the phosphorylation of the PI3K/Akt/mTOR pathway via PEDF. (a, b) The ratios of p-Akt/Akt protein and p-mTOR/mTOR protein were evaluated by western blotting. OM-MSCs^PEDFsiRNA^: knockdown of PEDF in OM-MSCs by transfecting PEDF-specific siRNA; MSCs^NCsiRNA^: OM-MSCs were transfected with normal control siRNA. Data were displayed as mean ± SD based on three independent experiments. ^∗∗∗^*p* < 0.001 compared with the normal group; ^#^*p* < 0.05, ^##^*p* < 0.01, and ^###^*p* < 0.001 compared with the OGD/R group; ^&^*p* < 0.05, ^&&^*p* < 0.01 compared with the OGD/R+OM-MSC group.

It is well documented that PEDF is associated with the activation of the PI3K/Akt/mTOR pathway; thus, the proteins levels of the PI3K/Akt/mTOR pathway were also examined. As shown in Figures 4(a) and 4(b), OGD/R treatment apparently decreased the ratio of phosphorylated Akt (p-Akt)/Akt and phosphorylated mTOR (p-mTOR)/mTOR, whereas both the ratio of p-Akt/Akt and p-mTOR/mTOR increased in the presence of OM-MSCs relative to those after OGD/R intervention. Knockdown of *PEDF* in OM-MSCs counteracted its capacity to upregulate the ratio of p-Akt/Akt and p-mTOR/mTOR in N2a cells after OGD/R (Figures 4(a) and 4(b)). These data implied that OM-MSCs may exhibit neuroprotection by activating the PEDF-PI3K/Akt/mTOR pathway.

### 3.6. OM-MSCs Reduced the GA Stress Response by Regulating the PI3k/Akt/mTOR Pathway in OGD/R-Injured N2a Cells

Accumulating evidence regarding the abrogation effect of OM-MSCs on the GA stress response following cerebral IRI drove us to investigate in depth the molecular clues involved in the neuroprotection of OM-MSCs. Therefore, the role of the PI3K/Akt/mTOR signaling pathway in OM-MSC-mediated neuroprotection was further investigated. The effects of OM-MSCs on the levels of the Akt/mTOR pathway were restricted by perifosine, a phosphorylation inhibitor of Akt. The use of rapamycin could offset the upregulation of p-mTOR/mTOR by OM-MSCs, with no effect on the ratio of p-Akt/Akt, implying that OM-MSCs may exhibit neuroprotection by activating the PI3K/Akt/mTOR pathway (Supplementary Figure S3A-B).

**Figure 5:**
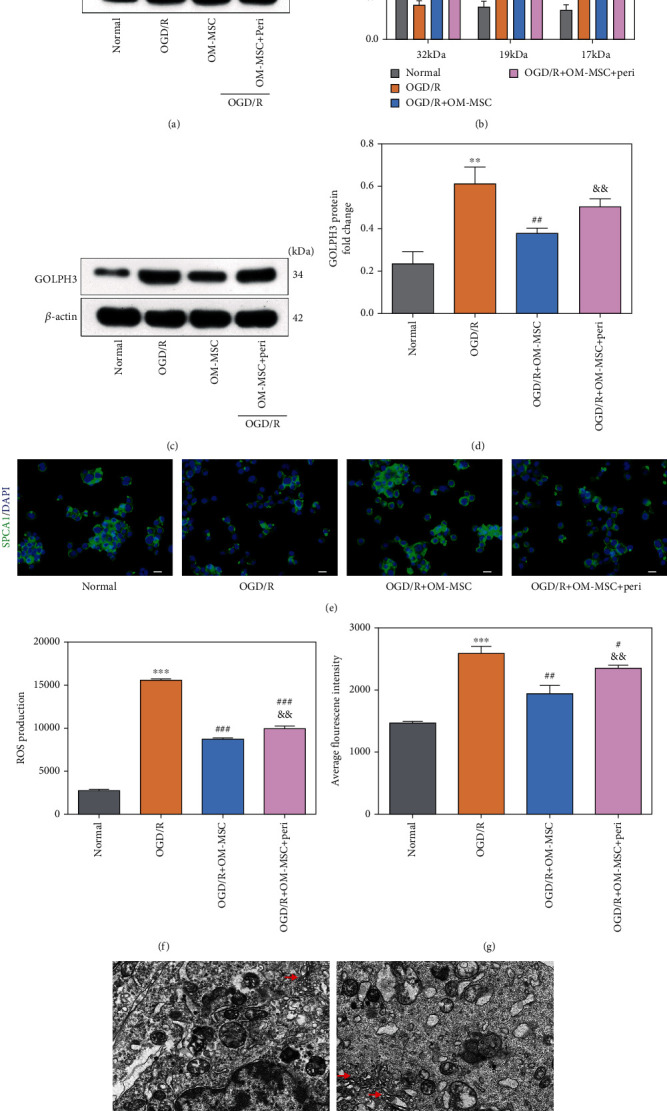
OM-MSCs reduced the GA stress response by regulating the PI3K/Akt/mTOR pathway in OGD/R-injured N2a cells. (a, b) The protein expression of caspase-3 was examined by western blotting. (c, d) The protein expression of GOLPH3 was determined by western blotting. (e) The representative image of SPCA1 immunofluorescence analysis (scale bar = 40 *μ*m). (f) The level of intracellular ROS was measured by flow cytometry analysis using an oxidation-sensitive fluorescent probe (DCFH-DA). (g) The concentration of intracellular Ca^2+^ in N2a cells was measured by flow cytometry analysis using a Fluo-3/AM kit. (h) The representative image of GA ultramicrostructure changes by using a transmission electron microscope (scale bar = 2.0 *μ*m). The GA was indicated by the red arrow. Peri: perifosine, added to the medium of N2a cells at the onset of reoxygenation (10 *μ*M). Data were displayed as mean ± SD based on three independent experiments. ^∗∗^*p* < 0.01, ^∗∗∗^*p* < 0.001 compared with the normal group; ^#^*p* < 0.05, ^##^*p* < 0.01, and ^###^*p* < 0.001 compared with the OGD/R group; ^&&^*p* < 0.01 compared with the OGD/R+OM-MSC group.

Subsequently, the indicators of the GA stress response were visualized after the PI3K/Akt/mTOR pathway was inhibited by perifosine. Perifosine partially neutralized the alleviation effect of OM-MSCs on the OGD/R-induced GA stress response, as evidenced by caspase-3 (Figures 5(a) and 5(b)), GOLPH3, and SPCA1 expression (Figures 5(c)–5(e)), ROS level, and intracellular Ca^2+^ concentration (Figures 5(f) and 5(g)). Furthermore, the application of perifosine abrogated the ability of OM-MSCs to prevent GA fragmentation caused by OGD/R insult (Figure 5(h)). Based on the existing findings, OM-MSCs diluted the GA stress response, at least partially, by regulating the PI3K/Akt/mTOR pathway following cerebral IRI injury.

### 3.7. OM-MSCs Suppressed OGD/R-Induced Autophagy by Activating the PI3k/Akt/mTOR Pathway in N2a Cells

**Figure 6:**
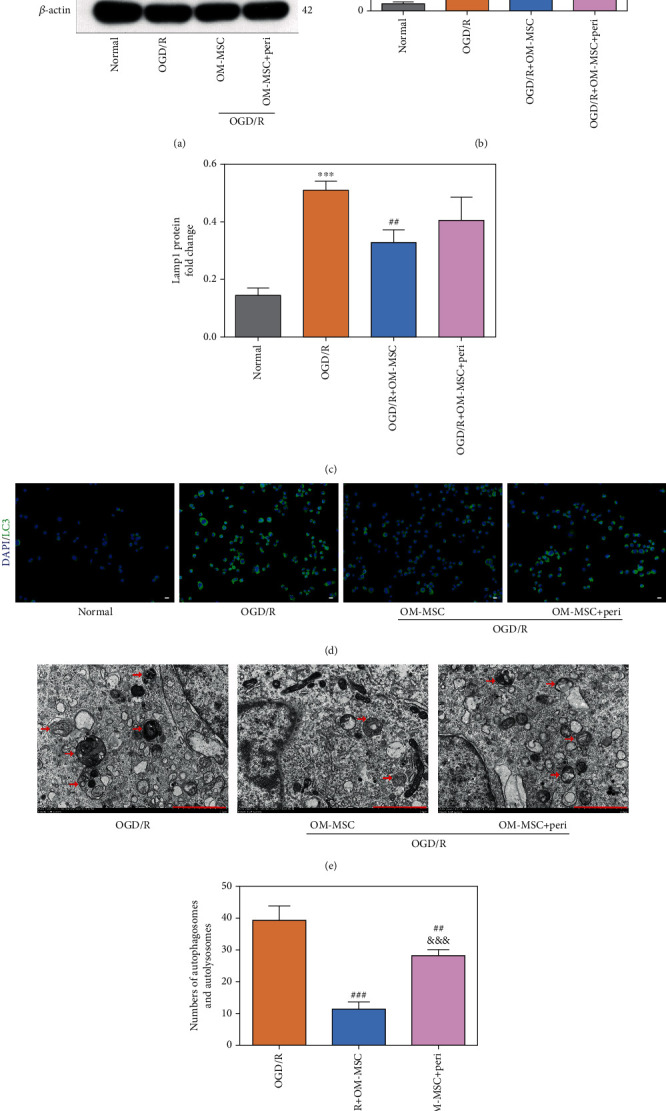
OM-MSCs inhibited OGD/R-induced autophagy by promoting the phosphorylation of the PI3K/Akt/mTOR pathway in N2a cells. (a–c) The ratio of LC3II/LC3I protein and the protein expression of Lamp1 were detected by western blotting. (d) The representative image of LC3 immunofluorescence analysis (scale bar = 50 *μ*m). (e, f) The autophagosome and autolysosome were observed by transmission electron microscopy (scale bar = 2.0 *μ*m, *n* = 5). The autophagosome and autolysosome were indicated by the red arrow. Peri: perifosine, added to the medium of N2a cells at the onset of reoxygenation (10 *μ*M). Data were displayed as mean ± SD based on three independent experiments. ^∗∗^*p* < 0.01, ^∗∗∗^*p* < 0.001 compared with the normal group; ^#^*p* < 0.05, ^##^*p* < 0.01, and ^###^*p* < 0.001 compared with the OGD/R group; ^&&^*p* < 0.01, ^&&&^*p* < 0.001 compared with the OGD/R+OM-MSC group.

Meanwhile, the autophagic biomarkers were also evaluated after the Akt/mTOR pathway was inhibited by perifosine because the activation of the PI3K/Akt/mTOR pathway is closely related to autophagy modulation [27]. As shown in Figures 6(a)–6(c), the ratio of LC3II/LC3I and the protein level of Lamp1 were significantly higher in the OGD/R group than in the normal group. Notably, OM-MSC intervention partially reversed the alterations induced by OGD/R. Furthermore, perifosine could partially restrict the downregulation of the LC3II/LCI ratio and Lamp1 protein level by OM-MSCs. Immunofluorescence analysis of LC3 further validated these findings (Figure 6(d)). At the same time, an electron microscope showed that OGD/R treatment led to an increase in the numbers of autophagosomes and autolysosomes, which could be inhibited by OM-MSC administration. Similarly, inhibition of Akt/mTOR by perifosine partially abolished the effect of OM-MSCs on the formation of autophagosomes and autolysosomes (Figures 6(e) and 6(f)). These results provided evidence that OM-MSCs were capable of modulating OGD/R-induced autophagy by activating the PI3k/Akt/mTOR pathway.

## 4. Discussion

It is well documented that GA dysfunction has been implicated in a wide range of human diseases [28], and the role of GA in oxidative stress-related damage has been studied extensively [5, 29]. Previous evidence suggests that oxidative stress caused by cerebral IRI contributes to the upregulation of GOLPH3, GA fragmentation, and intracellular Ca^2+^ concentration along with the downregulation of SPCA1, which is referred to as the GA stress response. More specifically, Li et al. reported that elevated GOLPH3 further induced GA fragmentation and activated downstream apoptosis and autophagy signals in OGD/R-treated N2a cells, which could be reversed by the silencing of GOLPH3 [30]. Another study demonstrated the essential role of SPCA1 in cytosolic Ca^2+^ regulation, which could be impaired by cerebral IRI [31]. Additionally, the GA stress response triggered by oxidative stress could in turn complicate oxidative stress, including disruption of oxidative phosphorylation, increased ROS generation, and promotion of lipid peroxidation, further emphasizing the importance of GA in oxidative stress [4, 32]. In the present study, the GA stress responses, including GOLPH3 upregulation, SPCA1 downregulation, ROS and LPO generation, Ca^2+^ overload, and GA fragmentation, were observed in OGD/R-treated N2a cells and MCAO rats, which was in line with previous findings.

The molecular and cellular mechanisms involved in the pleiotropic effects of MSCs on ischemic stroke have been widely explored in previous studies [14, 33], while investigations into the neuroprotective mechanisms of MSCs targeting GA are only commencing. Our previous study has disclosed that the neuroprotection of OM-MSCs against cerebral IRI was achieved partially through improving the expression and function of SPCA1 and reducing the edema and dissolution of the GA in neurons [21]. The present paper further displayed the ability of OM-MSCs to repress the elevation in GOLPH3 caused by cerebral IRI, providing a comprehensive interpretation regarding the mitigation effect of OM-MSCs on the GA stress response after cerebral IRI.

The available data suggest that PEDF exhibits neuroprotective properties, including anti-inflammatory and antioxidative, in disease models [34]. For instance, Sanchez et al. found that PEDF protected cortical neurons from oxidant injury by activating extracellular signal-regulated kinase (ERK) 1/2 and inducing Bcl-2 [35]. Qiu et al. demonstrated that PEDF prolonged cell activity during OGD by promoting proteasomal degradation of AMPK and reducing ATP production [36]. Another study suggested that PEDF possessed the ability to improve BBB integrity after cerebral ischemia [37]. However, evidence regarding the effects of PEDF on GA function is lacking. Here, based on a loss-of-function assay, we confirmed that OM-MSCs ameliorated the GA stress response following cerebral IRI partly through the secretion of PEDF.

As a negative regulatory element of autophagy induction, phosphorylation of the PI3K/Akt/mTOR signaling pathway is always impaired during cerebral ischemia/reperfusion [38, 39]. Moreover, several studies on the restorative effect of stem cells on the PI3K/Akt/mTOR pathway in ischemic stroke models with positive results have been reported [17, 40–43]. The reduction of p-Akt and p-mTOR induced by OGD/R insult was also observed in this study, and treatment with OM-MSCs partially restored the expression of p-Akt and p-mTOR in OGD/R-treated N2a cells. However, *PEDF*-silenced OM-MSCs exhibited a reduced neuroprotective effect on the activation of the PI3k/Akt/mTOR pathway, probably indicating the direct regulation impact of OM-MSC-derived PEDF on this pathway.

It is well described that exogenous PEDF interacts with specific receptors located on the cell membrane and then triggers the downstream signaling pathways. At present, two subtypes of receptors have been identified: the PEDF receptor (PEDF-R) and laminin receptor (LR) [44]. The protective effect of PEDF against IRI in a PEDFR-dependent manner was recently documented [45, 46]. Interestingly, in one such study, the authors found that PEDF increased the phosphorylation of Akt in the rat ischemic myocardium [45]. Therefore, OM-MSC-derived PEDF probably upregulated the phosphorylation of Akt in OGD/R-injured N2a cells by interacting with PEDF-R.

More importantly, the attenuation effect of OM-MSCs on the GA stress response could be partially abolished by the Akt inhibition, using perifosine, further demonstrating that the modulation of the PI3K/Akt/mTOR pathway by OM-MSCs participated in the regulation of the GA stress response after cerebral IRI.

**Figure 7:**
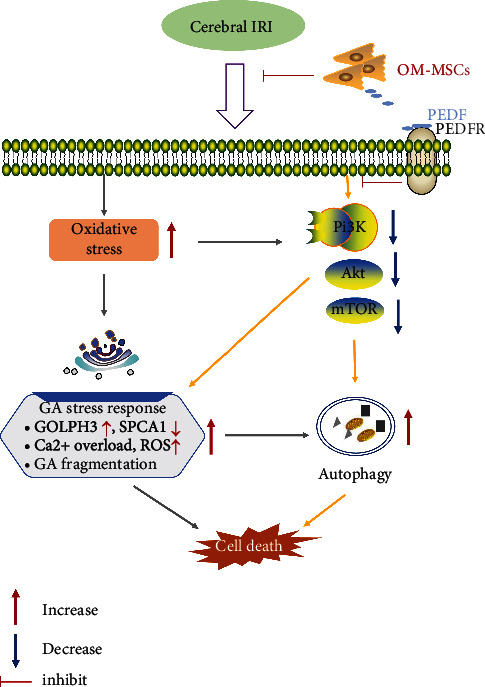
Schematic diagram showing the proposed signaling pathways involved in the neuroprotective effects of OM-MSCs on the GA stress response following cerebral ischemia/reperfusion injury (IRI).

Autophagy is a dynamic process of self-degradation of intracellular components mediated by lysosomal enzymes [47] and exerts a biphasic effect on ischemic stroke [48]. Appropriate autophagy contributes to the removal of damaged tissues, while excessive autophagy exacerbates brain injury under ischemic conditions, and the inhibition of autophagy confers neuroprotection against cerebral IRI [49, 50]. Here, the levels of autophagy-related proteins and the number of autophagosomes and autolysosomes were upregulated in the OGD/R-treated N2a cells, accompanied by apoptosis and GA stress responses, indicating that the elevated autophagy activity was involved in the process of N2a cell OGD/R injury. Subsequent findings suggested that OM-MSCs possessed the ability to downregulate the levels of autophagy following cerebral IRI, which were in line with prior data regarding other types of MSCs [41, 51]. Moreover, this ability could be partially neutralized by intervention with perifosine. The present study showed that OM-MSCs mitigated the GA stress response, suppressed autophagy, and eventually inhibited apoptosis partially through the PEDF-PI3K/Akt/mTOR pathway (Figure 7).

The interplay between the GA stress response and autophagy in ischemic stroke is intricate. Previous evidence showed that the GA stress response could promote the progression of autophagy, wherein the upregulation of GOLPH3 stimulated the generation of ROS [30], a forceful activator of autophagy. Moreover, GOLPH3 was also able to inhibit the Akt/mTOR signaling pathway, thereby inducing autophagy [52, 53]. The inhibition of GA stress response by silencing GOLPH3 remarkably downregulated autophagy [30]. However, the impact of suppressing autophagy on the GA stress response following cerebral IRI remains unclear. Therefore, further defining the complicated crosstalk between the GA stress response and autophagy is necessary to completely elucidate the key role of GA stress response in cerebral IRI.

Nevertheless, there are still some limitations regarding the present research, which are expected to be addressed in subsequent studies. First, only the 18-point mNSS was used to evaluate neurological deficit in this study, but the mNSS might not be optimal for the assessment of neurobehavioral function in experimental stroke [54]. More behavior test should be performed to identify the MCAO model and assess the neurological deficit, such as the rotarod test, cylinder test, adhesive label test, and Montoya's staircase test. Second, the establishment of gene-knockout rats is of great significance for a more integrated interpretation of the correlation between the GA stress response and autophagy in ischemic stroke. Third, despite the application of drugs or siRNA, OM-MSCs still exhibited the capacity to ameliorate part of the GA stress response and inhibit autophagy in ischemic stroke models. This outcome is largely due to the potent paracrine activity of OM-MSCs. The ability to penetrate the blood-brain barrier, low immunogenicity, and properties similar to MSCs make MSC-derived extracellular vesicles (EVs) a better source of stroke therapies compared to MSCs [55]. Accordingly, as the OM-MSC-derived EVs have also been recently identified [56], the exploration of the mechanism of OM-MSC-derived EVs on the GA stress response in ischemic stroke would be attractive.

## 5. Conclusion

In summary, the present data demonstrated that OM-MSCs downregulated GOLPH3 expression, upregulated SPCA1 expression, inhibited ROS production, and alleviated intracellular Ca^2+^ overload in ischemic stroke models via secretion of PEDF. The involvement of the PI3K/Akt/mTOR pathway and autophagy in the neuroprotection of OM-MSCs was further investigated. These findings could provide novel insights into the mechanisms of OM-MSCs in the treatment of ischemic stroke and other conditions.

## Data Availability

The authors declare that all data supporting the findings of this study are available within the paper and its supplementary materials.

## Supplementary Materials

Supplementary 1 Supplementary Figure S1: the characterization of OM-MSCs. A. The morphology of OM-MSCs in the third passage. Scale bar = 200 *μ*m. B. The flow cytometry assessed the immunophenotypic marker of OM-MSCs.

Supplementary 2 Supplementary Figure S2: the efficiency of *PEDF* knockdown in OM-MSCs. A. The mRNA expression of PEDF in OM-MSCs after transfection of PEDF siRNA was measured by qRT-PCR. B. The protein expression of PEDF protein in the supernatant of OM-MSCs after transfection of PEDF siRNA was evaluated by ELISA. C-D. The protein expression of PEDF in OM-MSCs after transfection of PEDF siRNA was examined by western blotting. NC siRNA: normal control siRNA. Data were displayed as mean ± SD based on three independent experiments. ^∗^*p* < 0.01, ^∗∗^*p* < 0.01, and ^∗∗∗^*p* < 0.001 compared with the normal group.

Supplementary 3 Supplementary Figure S3: A-B. The ratios of p-Akt/Akt protein and p-mTOR/mTOR protein in N2a cells were evaluated by western blotting. Peri: perifosine, added to the medium of N2a cells at the onset of reoxygenation (10 *μ*M). Rapa: rapamycin, added to the medium of N2a cells at the onset of reoxygenation (50 nM). Data were displayed as mean ± SD based on three independent experiments. ^∗∗^*p* < 0.01, ^∗∗∗^*p* < 0.001 compared with the normal group; ^#^*p* < 0.05, ^###^*p* < 0.001 compared with the OGD/R group; ^&^*p* < 0.05, ^&&^*p* < 0.01 compared with the OGD/R+OM-MSC group.
