# Supplementary figures and images for: OM-MSCs Alleviate the Golgi Apparatus Stress Response following Cerebral Ischemia/Reperfusion Injury via the PEDF-PI3K/Akt/mTOR Signaling Pathway
Source: Oxid Med Cell Longev. 2021 Nov 13;2021:4805040. doi: 10.1155/2021/4805040 (PMC8606042; doi:10.1155/2021/4805040)

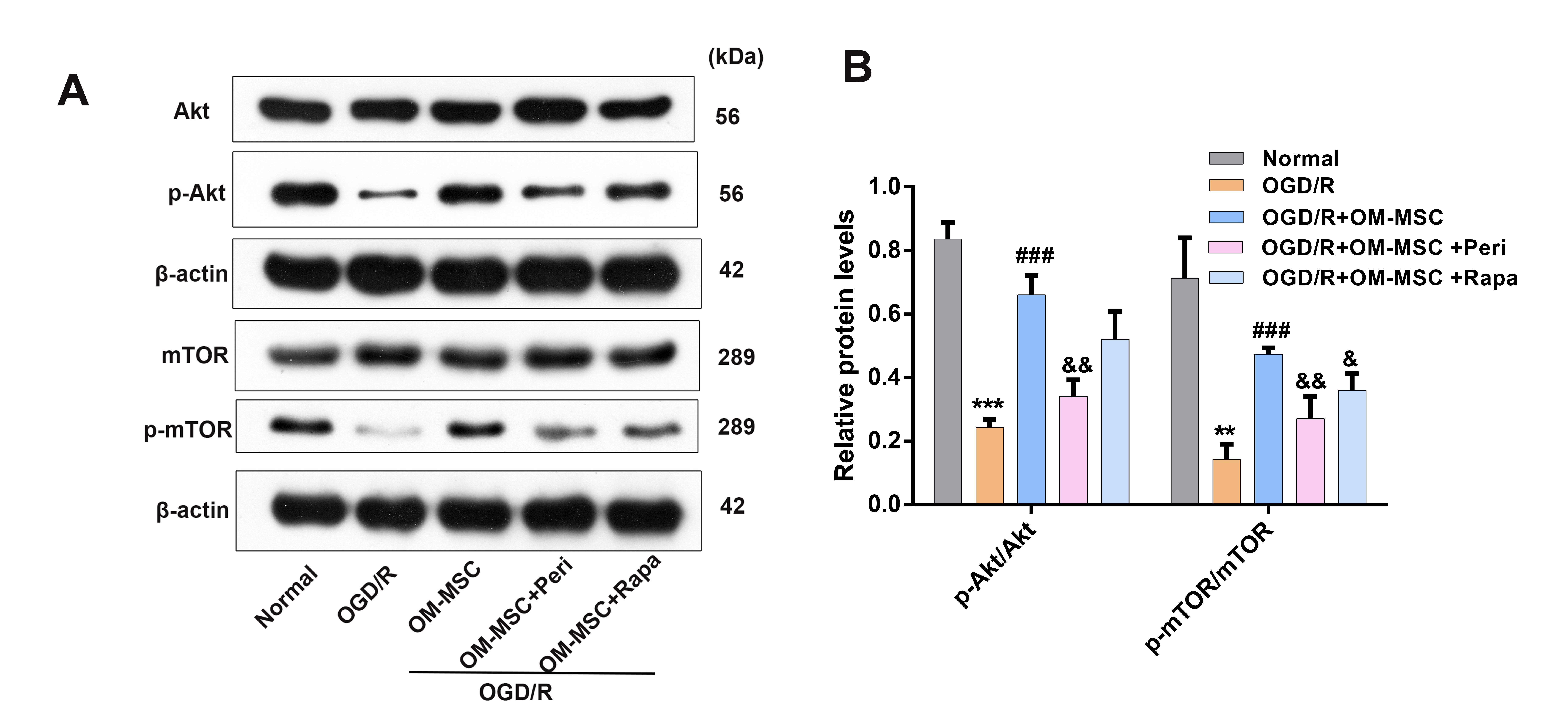

Supplement: Supplementary 3 — Supplementary Figure S3: A-B. The ratios of p-Akt/Akt protein and p-mTOR/mTOR protein in N2a cells were evaluated by western blotting. Peri: perifosine, added to the medium of N2a cells at the onset of reoxygenation (10 μM). Rapa: rapamycin, added to the medium of N2a cells at the onset of reoxygenation (50 nM). Data were displayed as mean ± SD based on three independent experiments. ∗∗p < 0.01, ∗∗∗p < 0.001 compared with the normal group; #p < 0.05, ###p < 0.001 compared with the OGD/R group; &p < 0.05, &&p < 0.01 compared with the OGD/R+OM-MSC group. [file 4805040.f3.jpg]
